# Evolutionary Relationships between Bat Coronaviruses and Their Hosts

**DOI:** 10.3201/eid1310.070448

**Published:** 2007-10

**Authors:** Jie Cui, Naijian Han, Daniel Streicker, Gang Li, Xianchun Tang, Zhengli Shi, Zhihong Hu, Guoping Zhao, Arnaud Fontanet, Yi Guan, Linfa Wang, Gareth Jones, Hume E. Field, Peter Daszak, Shuyi Zhang

**Affiliations:** * East China Normal University, Shanghai, People’s Republic of China; † Hebei Normal University, Hebei, People’s Republic of China; ‡ Chinese Academy of Sciences, Beijing, People’s Republic of China; § University of Georgia, Athens, Georgia, USA;; ¶ Wuhan Institute of Virology, Wuhan, People’s Republic of China; # Shanghai Institutes of Biological Sciences, Shanghai, People’s Republic of China; ** Insitut Pasteur, Paris, France; †† University of Hong Kong, Hong Kong Special Administrative Region, People’s Republic of China; ‡‡ Australian Animal Health Laboratory, Geelong, Victoria, Australia; §§ University of Bristol, Bristol, United Kingdom; ¶¶ Department of Primary Industries and Fisheries, Yeerongpilly, Queensland, Australia; ## Consortium for Conservation Medicine, New York, New York, USA; 1 These authors contributed equally to this study.

**Keywords:** Phylogeny, coronaviruses, bats, SARS, phylogeography, evolution, host shifts, research

## Abstract

Recent studies have suggested that bats are the natural reservoir of a range of coronaviruses (CoVs), and that rhinolophid bats harbor viruses closely related to the severe acute respiratory syndrome (SARS) CoV, which caused an outbreak of respiratory illness in humans during 2002–2003. We examined the evolutionary relationships between bat CoVs and their hosts by using sequence data of the virus RNA-dependent RNA polymerase gene and the bat cytochrome b gene. Phylogenetic analyses showed multiple incongruent associations between the phylogenies of rhinolophid bats and their CoVs, which suggested that host shifts have occurred in the recent evolutionary history of this group. These shifts may be due to either virus biologic traits or host behavioral traits. This finding has implications for the emergence of SARS and for the potential future emergence of SARS-CoVs or related viruses.

## Abstract 2

Host shifting has occurred among *Rhinolophus* spp., with potential implications for emergence of SARS.

Severe acute respiratory syndrome (SARS) emerged in November 2002 in southern People’s Republic of China (*1*), and a SARS coronavirus (SARS-CoV) was identified as the etiologic agent (*2*). These events and the identification of SARS-CoV in animals associated with the wildlife trade in southern China (*3*) have led to a rapid resurgence of interest in CoVs of different origins. This resurgence led to discovery of 2 novel human CoVs (*4**,**5*); identification of SARS-like CoVs in horseshoe bats (*Rhinolophus macrotis*, *R*. *ferrumequinum*, *R*. *pearsoni*, and *R*. *sinicus*) (*6**,**7*); and identification of other CoVs in bat species (*R*. *sinicus*, *R*. *ferrumequinum*, *Miniopterus magnater* [*M*. *magnater* has been misidentified as *M*. *schreibersi* (*8*) in reports on SARS-like CoV], *Pipistrellus abramus*, *P*. *pipistrellus*, *Tylonycteris pachypus*, *Myotis ricketti*, and *Scotophilus kuhlii*) (*7**,**9**–**12*). However, evolutionary relationships among these CoVs and their bat hosts have not been examined.

Studies in species other than bats have examined host-virus phylogeny and identified coevolutionary relationships (*13**–**16*) or incongruous phylogenetic patterns (*17*). These findings suggest recent pathogen host shifts (defined as interspecies transmission followed by establishment and long-term persistence in the new host species [*18*]). Other studies have demonstrated that the relationship between viral phylogeny and geographic location and identification hosts (viral phylogeography [*19*]) can yield information on the origin of emerging zoonoses (*19**,**20*).

Knowing the high genetic diversity of bat CoVs, we carried out a systematic phylogenetic study of the viruses and their hosts to examine evolutionary relationships between bat CoVs and bats. The aim was to further investigate the origin of SARS-like CoVs and SARS. Our results suggest host-pathogen divergence and host shifts in the recent evolutionary history of these viruses and their hosts. We discuss host behavioral traits and viral traits that might have given rise to these patterns and comment on the implications of our findings for the emergence of SARS-CoV.

## Materials and Methods

### CoV Sequences

Only CoVs from bats were included in this study. We used gene sequences that Tang et al. obtained from 10 bat species (*R*. *sinicus*, *R*. *ferrumequinum*, *R*. *macrotis*, *R*. *pearsoni*, *M*. *magnater*, *P*. *abramus*, *P*. *pipistrellus*, *T*. *pachypus*, *S*. *kuhlii*, and *Myotis ricketti*) (*10*). An additional 57 bat CoV sequences available in GenBank were also included in this analysis.

### Bat Mitochondrial Cytochrome b (*cyt b*) Gene Sequences

Tissue samples were obtained from 3-mm wing membrane biopsy specimens from wild bats, which had been caught in 9 provinces of China, that had been preserved in 99% ethanol. Genomic DNA was extracted by using the DNeasy Tissue Kit (QIAGEN, Valencia, CA, USA) and stored at –20°C. We used complete *cyt b* sequences of *R*. *ferrumequinum*, *P*. *abramus*, and *P*. *pipistrellus*, which have recently been published and are available in Genbank. We generated *cyt b* sequences from *M*. *magnater* (n = 4), *T*. *pachypus* (n = 3), *R*. *macrotis* (n = 2), *R*. *pearsoni* (n = 2), *R*. *sinicus* (n = 2), *S*. *kuhlii* (n = 1), and *Myotis* *ricketti* (n = 1).

PCR mixtures were prepared in 50-μL volumes containing 25 μL 2× EXTaq DNA polymerase (TaKaRa, Kyoto, Japan). Two pairs of primers, Bat_Cytb_1 (5′-TAG AAT ATC AGC TTT GGG TG-3′) (*21*) with Bat_Cytb_2 (5′-AAA TCA CCG TTG TAC TTC AAC-3′) (*21*), and Bat_Cytb_2 with BAT15R (5′-TCA GCT TTG GGT GTT GAT GG-3′) (*22*), were used because of amplification specificity of certain primers in some species. Amplification was conducted at an initial denaturing temperature at 94°C for 30 s; 34 cycles of denaturation at 94°C for 30 s, annealing at 55°C for 30 s, and extension at 72°C for 90s; and a final extension at 72°C for 10 min. The PCR samples were then stored at 4°C. The complete mitochondrial *cyt b* gene (1,140 bp) was amplified and sequenced. These sequences have been submitted to GenBank and accession numbers are shown in the Table.

**Table:** Bat host species analyzed and their typical roosting sites*

Host species	No. sampled (no. RT positive)	Roosting sites	GenBank accession no.†	Location
*Miniopterus magnater*	365 (56)	Caves	EF517305‡	Hubei, China
			EF517306‡	Yunnan, China
			EF517308‡	Hainan, China
			EF517307‡	Hong Kong, China
*Myotis ricketti*	76 (14)	Caves	EF517316‡	Beijing, China
			AB106608	Guangdong, China
			AJ504452	Laos
*Pipistrellus abramus*	55 (18)	Old buildings	AB085739	Japan
			AJ504448	Taiwan
*P. pipistrellus*	27 (6)	Old buildings	AJ504443	Taiwan
*Scotophilus kuhlii*	43 (5)	Under palm leaves	EF543860‡	Hainan
				
*Tylonycteris pachypus*	35 (6)	Internodes of bamboo	EF517315‡	Guangdong, China
			EF517313‡	Guangxi, China
			EF517314‡	Hong Kong, China
*Rhinolophus ferrumequinum*	49 (5)	Caves	DQ297575	Yunnan, China
			DQ351847	Jilin, China
			AB085725	Japan
*R. macrotis*	8 (1)	Caves	EF517311‡	Yunnan, China
			EF517312‡	Yunnan, China
*R. pearsoni*	78 (4)	Caves	EF517309‡	Guizhou, China
			EF517310‡	Yunnan, China
			DQ297587	Sichuan, China
*R. sinicus*	125 (24)	Caves	EF517303‡	Guizhou, China
			EF517304‡	Guizhou, China

*Bats roosting in caves usually have higher population densities and greater chances of physical contact. RT, reverse transcription. †For mitochondrial cytochome b gene sequences. ‡These sequences were provided by our laboratory.

### Phylogenetic Analysis of CoV Sequences

**Figure 1:**
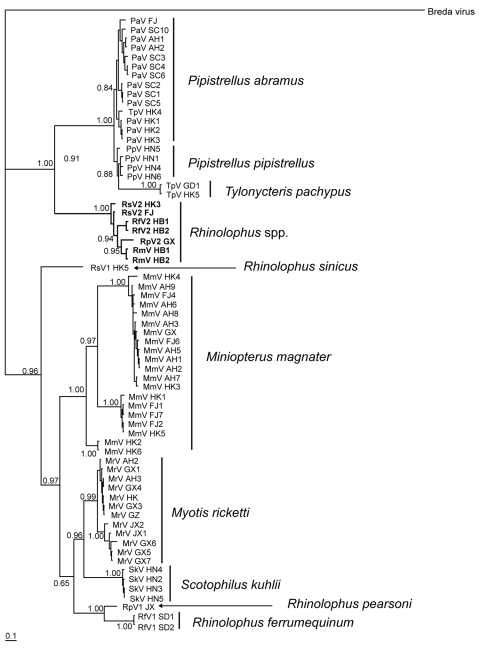
Phylogram of bat coronaviruses based on the 440-bp RNA-dependent RNA polymerase gene region. Methods used are described in the text. Values to the left of branches are Bayesian posterior probabilities. Scale bar at the lower left indicated 0.1 nucleotide substitutions per site. **Boldface** branches indicate severe acute respiratory syndrome–like coronaviruses, and species names to the right of lineages indicate putative reservoir host(s). Pa, *Pipistrellus abramus*; Tp, *Tylonycteris pachypus*; Pp, *P*. *pipistrellus*; Rs, *Rhinolophus sinicus*; Rf, *R*. *ferrumequinum*; Rp, *R*. *pearsoni*; Rm, *R*. *macrotis*; Mm, *Miniopterus magnater*; Mr, *Myotis ricketti*; Sk, *Scotophilus kuhlii*. Sequences obtained from GenBank were as follows: DQ412043 isolated from *R*. *macrotis* in Hubei Province (HB); DQ412042 isolated from *R*. *ferrumequinum* in HB; DQ071615 isolated from *R*. *pearsoni* in Guangxi Province (GX); DQ022305, DQ084199, DQ084200, DQ249213, and DQ249235 isolated from *R*. *sinicus* in Hong Kong (HK); DQ249214, DQ249215, DQ249216, DQ249217, and DQ074652 isolated from *T*. *pachypus* in HK; DQ249218, DQ249219, and DQ249221 isolated from *Pipistrellus abramus* in HK; DQ249224 isolated from *Myotis ricketti* in HK; and DQ249226, DQ666337, DQ666339, DQ666340, DQ249228, and DQ666338 isolated from *M*. *magnater* in HK. FJ, Fujian Province; SC, Sichuan Province; AH, Anhui Province; HN, Hainan Province; GD, Guandong Province; JX, Jiangxi Province; SD, Shandong Province.

For virus phylogeny studies, sequences from a 440-bp fragment of the RNA-dependent RNA polymerase (*RdRp*) gene, which is highly conserved among different CoVs, were obtained and analyzed (*10*). Multiple alignments of the 440-bp *RdRp* partial sequence of bat CoVs were conducted in ClustalX version 1.81 (*23*). Bayesian analyses were conducted with MrBayes version 3.1.2 (*24*). Neighbor-joining analyses (with the Jukes-Cantor model) were used to validate the Bayesian result in MEGA3 (*25*). A total of 67 unique CoV sequences (Figure 1) were analyzed with MrBayes version 3.1.2 in the generalized time reversible model of evolution as determined by the Akaike Information Criterion in MODELTEST version 3.7 (*26*). Four consecutive Metropolis-coupled Markov chain Monte Carlo computations were run for 2 million generations, with trees sampled every 100 generations. Initial trees were random. On the basis of stabilization of preliminary runs, the first 3,000 trees were discarded before generation of the consensus tree. The Bayesian consensus tree was rooted to Breda virus (AY427798), a related CoV (Figure 1).

### Phylogenetic Analyses of Bat *cyt b* Gene Sequences

For bat phylogeny, we used the complete mitochondrial *cyt b* gene to construct maximum likelihood (ML) and Bayesian phylograms. The *cyt b* sequence data were aligned by using ClustalX version 1.81 as above. ML analysis was performed by using PAUP* version 4.0b (*27*). The most appropriate substitution model (generalized time reversible + Γ + I) with the parameters matrix = 0.4835 × 9.6665 × 0.3815 × 0.2973 × 7.1418, base frequency = 0.3576 × 0.3420 × 0.0748, rates = gamma, shape = 0.6008, and proportion of invariable sites unable to accept substitutions = 0.4078 for ML and subsequent Bayesian analysis was calculated by using the program Modeltest 3.7 (*26*). We used heuristic searches (10 replicates, random addition of taxa, with tree bisection and reconnection branch swapping), followed by 100 bootstrap iterations for robustness of the ML tree. Bayesian analysis was also used to construct a tree with 4 simultaneous Markov chains for 1 million generations. Trees were sampled every 20 generations, and the first 5,000 trees were discarded before the consensus tree was made (on the basis of practical values of stabilizing likelihood).

### Genetic Diversity among Bats and CoVs

We compared the genetic diversity of CoVs isolated from rhinolophids and vespertilionids and the corresponding diversity among bat taxa by using the index of nucleotide diversity (π) described by Nei (*28*) in Arlequin version 3.1 (*29*). Analyses were performed on uncorrected pairwise genetic distances between sequences.

## Results

**Figure 2:**
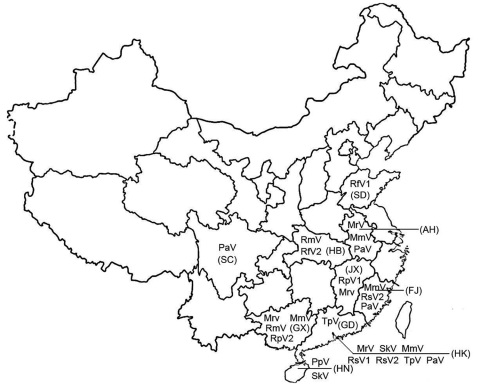
Distribution of coronaviruses isolated in the People’s Republic of China. RsV, detected in *Rhinolophus sinicus*; PaV, detected in *Pipistrellus abramus*; TpV, detected in *Tylonycteris pachypus*; RfV, detected in *R*. *ferrumequinum*; RmV, detected in *R*. *macrotis*; PpV, detected in *P*. *pipistrellus*; SkV, detected in *Scotophilus kuhlii*; MrV, detected in *Myotis ricketti*; RpV, detected in *R*. *pearsoni*; MmV, detected in *Miniopterus magnater*; MpV, detected in *M*. *pusillus*. Abbreviations for provinces are shown in parentheses. SC, Sichuan Province; AH, Anhui Province; FJ, Fujian Province; HN, Hainan Province; GD, Guangdong Province; HB, Hubei Province; GX, Guangxi Province; SD, Shandong Province; JX, Jiangxi Province; HK, Hong Kong Special Administrative Region, People’s Republic of China.

By combining information derived from the phylogram of bat CoVs, together with data on the geographic origin of viruses, we were able to describe the phylogeographic distributions for known CoVs from bats in China (Figures 1, 2; Table). Bat SARS-like CoVs formed a monophyletic clade. Species-specific host restriction was found for CoVs in 4 of 7 bats species (*Myotis ricketti*, *M*. *magnater*, *P*. *abramus*, and *T*. *pachypus*) sampled from >1 geographic location, and these clustered with high Bayesian posterior probability. Overall phylogenetic relationships between virus lineages were similar across our analyses, and well-supported genetic structure was observed within some CoV lineages. For example, CoVs isolated from *M*. *magnater* were monophyletic but formed 3 well-supported clades with no evidence of geographic structure (Bayesian posterior probability [PP] = 1.0 for each). A similar pattern was apparent in CoVs from *Myotis ricketti*, which formed 2 geographically overlapping independent clades (PP = 0.99 and 1.0, respectively). One *T*. *pachypus* was infected by a virus that clustered with moderate statistical support (PP = 0.91) within the larger clade associated with *P*. *abramus*, which indicated a potential interspecies transmission event or recent evolutionary host shift (defined as interspecies transmission followed by establishment and long-term persistence in the new host species [*18*]) (Figure 1).

**Figure 3:**
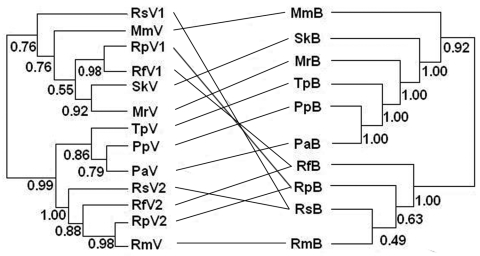
Phylogenetic relationships between coronaviruses (left) and their host bat species added for reference (right). Abbreviations on both sides denote viruses harbored by bats (marked as V on the left) and bats (marked as B on the right). Rs, *Rhinolophus sinicus*; Mm, *Miniopterus magnater*; Sk, *Scotophilus kuhlii*; Rp, *R*. *pearsoni*; Mr, *Myotis ricketti*; Rf, *R*. *ferrumequinum*; Tp, *Tylonycteris pachypus*; Pp, *Pipistrellus pipistrellus*; Pa, *P*. *abramus*; Rm, *R*. *macrotis*. Values below branches are Bayesian posterior probabilities. Although some of these values are low, our analysis demonstrated a pathway for future study (*28*). Lines between the 2 trees were added to help visualize virus and host sequence congruence or incongruence.

**Figure 4:**
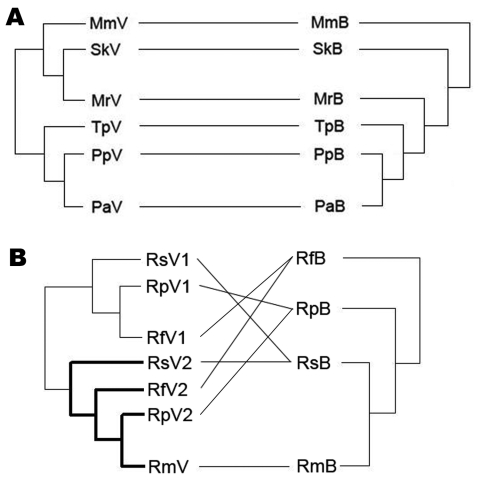
Phylogenetic relationships between coronaviruses (CoVs) (left) and bats (right) in the A) Vespertilionidae and B) Rhinolophidae. Abbreviations on both sides denote viruses harbored by bats (marked as V on the left) and bats (marked as B on the right). Mm, *Miniopterus magnater*; Sk, *Scotophilus kuhlii*; Mr, *Myotis ricketti*; Tp, *Tylonycteris pachypus*; Pp, *Pipistrellus pipistrellus*; Pa, *P*. *abramus*; Rs, *Rhinolophus sinicus*; Rf, *R*. *ferrumequinum*; Rp, *R*. *pearsoni*; Rm, *R*. *macrotis*. **Boldface** branches in panel B contain severe acute respiratory syndrome–like CoVs reported. Lines between bat and virus trees were added to help visualize congruence or incongruence. Although this figure implies differences in propensity for host shifts between these families, all but 1 of the vespertilionid CoVs are from different genera, whereas all rhinolophid CoVs are from the same genera, which make meaningful comparisons difficult. Overall mean genetic differences are much greater between vespertilionid species than between rhinolophid species.

Phylograms of host sequences were also constructed and were essentially of the same topology with high support whether derived by using MrBayes version 3.1.2 or MEGA3 (data not shown). When we mapped host phylogram to virus, virus phylogeny did not always track host phylogeny (Figure 3). When separate host-virus phylograms were constructed for the 2 bat families (Verspertilionidae and Rhinolophidae), different corresponding relationships were evident. Verspertilionids and their CoVs showed phylogenetic congruence, and rhinolophids and their CoVs showed incongruous phylogenies (Figure 4).

We found evidence for evolutionarily divergent relationships for some vespertilionid viruses and their hosts when analyzed at the family scale (Figure 4, panel A). For example, divergence between viruses harbored by *P*. *pipistrellus* and *P*. *abramus* is congruent with their hosts. The divergence among other viruses was incongruent with divergence of host species, e.g., those from *S*. *kuhlii* and *Myotis ricketti*.

Rhinolophid bats and their viruses were analyzed at a different taxonomic scale (within genus). In this co-phylogeny, viral host shifts were the evident virus-host feature (Figure 4, panel B). Except for *R*. *macrotis*, all rhinolophidae bats had 2 distinct lineages of CoVs, and host shifts were found among viruses carried by *R*. *ferrumequinum*, *R*. *pearsoni*, and *R*. *sinicus*.

Genetic diversity of CoVs harbored by rhinolophids and vespertilionids was similar (vespertilionids π = 0.27 ± 0.13; rhinolophids π = 0.25 ± 0.13). In contrast, genetic diversity of *cyt b* sequences from bats was much higher among the vespertilonids (π = 0.17 ± 0.007) than among the rhinolophids (π = 0.09 ± 0.006).

## Discussion

CoVs sequenced from different bats of the same species clustered together, even when bats were collected in locations 1,000–2,000 km apart. This pattern was found for CoVs from *P*. *abramus*, *T*. *pachypus*, *Myotis ricketti*, and *M*. *magnater*. Bats of the genus *Miniopterus* are known to migrate long distances (*30*), which explains why the phylogeny of viruses isolated from *M*. *magnater* sampled in distant places (Guangxi, Anhui, Fujian, and Hong Kong) lacks geographic structure. In nonmigrating species such as bats of the genera *Pipistrellus* and *Tylonycteris*, intimate physical contact of bats in same cave or the same bamboo roost site, as well as periodic exchange of bats among neighboring colonies, may facilitate virus transmission among populations.

Despite the co-roosting of many bats species, we found little evidence of host shifts for some viruses. For example, CoVs from *M*. *magnater* and *Myotis ricketti* sampled in the same cave in Guangxi were divergent, although sample size was limited. Although *Myotis ricketti* has a closer phylogenetic relationship with *T*. *pachypus*, *P*. *pipistrellus*, and *P*. *abramus* than with *M*. *magnater* and *S*. *kuhlii*, its behavior and habits are closer to those of the last group. For example, *Myotis ricketti* and *S*. *kuhlii* bats roosts in caves (although *S*. *kuhlii* also roosts under palm leaves), whereas *T*. *pachypus* roosts inside bamboo and *P*. *abramus* roosts almost entirely in old buildings. Thus, it seems plausible that the close phylogenetic relationship between viruses harbored by *Myotis ricketti* and *S*. *kuhlii* reflects the similar behavior and ecology of their hosts.

The phylogenetic and phylogeographic associations we found suggest that there may be a coevolutionary relationship between some bat CoVs and their hosts. For example, sister taxa within the genus *Pipistrellus* independently maintained 2 distinct viruses that share a most recent common ancestor. A similar relationship was apparent among the viruses of some closely related genera (e.g., *Pipistrellus* and *Tylonycteris*), whereby divergence of each genus was mirrored by divergence in viral phylogeny. However, viruses are usually thought to have evolved more recently than their hosts (*31*). Thus, apparent coevolutionary patterns may reflect either a high frequency of host shift among closely related bat species or simultaneous lineage splitting of hosts and viruses. Host shifts among related bats might be favored by a variety of mechanisms, including preadaptation to overcome immune defenses or greater rates of interspecific contact relative to distantly related bat species. Phylograms with better resolution would enable statistical comparison of phylogenetic congruence and estimation of divergence times.

In the vespertilionids, close phylogenetic concordance between host and virus suggests a close, possibly evolutionarily divergent relationship. However, there are different scales of comparison between the Vespertillionidae, in which all but 1 CoV came from separate genera, and the Rhinolophidae, in which we examined a co-phylogeny of multiple species within 1 genus. Genetic diversity in the vespertilionids sampled was nearly double that of the rhinolophids, which was probably due to the greater number of species sampled and their broader taxonomic range. Despite this greater genetic diversity among vespertilionid bat hosts, the genetic diversity of CoVs did not differ between vespertilionids and rhinopholids. This diversity suggests that vespertilionids may maintain undiscovered CoVs or that rhinolophids might harbor disproportionate CoV diversity relative to diversity of their genus. We propose that future work may identify more vespertilionid bat CoVs, which would enable an accurate comparison of propensity for host shifts within this group.

In the rhinolophids, the host phylogram demonstrated genetic divergence between *R*. *ferrumequinum* and other species, as shown by the division of *Rhinolophus* bats into 2 groups. Each of these groups harbors CoVs from 2 clusters (SARS-like CoVs and other CoVs), which suggests multiple introductions of CoVs into these species.

Lack of concordance between phylogenies of rhinolophid bats and their CoVs can be interpreted as evidence for host shifts between bats of the genus *Rhinolophus*. Different species of *Rhinolophus* are often observed roosting inside the same cave, which facilitates virus transmission between species. However, the degree of host shifting of rhinolophid bat CoVs may not be particularly high relative to other genera of bats. This observation will be clarified when a greater diversity of CoVs from other bat genera is reported and the sequences are analyzed. These requirements support the need for further research on bat viruses (*32**,**33*).

Host-shifting within the genus *Rhinolophus* would likely be promoted if these bats shed CoVs in a way that makes them more available to other *Rhinolophus* spp.; had behavioral traits that lead to increased contact with other *Rhinolophus* spp.; or if CoVs harbored by these bats have structural, biologic, or other traits that make them more readily transmitted to other *Rhinolophus* spp. Two lines of evidence suggest that host traits are the most parsimonious explanation for host shifts within the genus *Rhinolophus*. First, SARS-like CoVs and other rhinolophid CoVs (RfV1 and RpV1) show evidence of interspecies transmission. Second, CoVs from other bat groups that are phylogenetically much closer to RfV_1_ and RpV_1_ than to the SARS-like CoVs do not show evidence of successful host shifts. Thus, the ability to jump hosts is unlikely to be a strictly viral trait.

The phylogeography of bat CoVs suggests that the bat SARS-like CoVs form a monophyletic clade that is both phylogenetically distinct from other bat CoVs and geographically isolated. Although we acknowledge that this interpretation may be limited by sample size, it may also indicate that rhinolophid bats, the hosts of a cluster of SARS-like CoVs within which human and civet SARS CoV nestle phylogenetically (*6**,**7*), are more likely to foster the host shifts of CoVs than are other bat species. The potential for close contact between bats, civets, and humans in the wildlife trade in southern China, coupled with a possible propensity of these bats to foster CoV host-shifts, could explain SARS-like CoVs as the source of SARS-CoV. This potential supports molecular results on bat CoVs that suggest a recent host shift from bats to civets or other animals and humans (*34*). Such host shifts may indicate a risk posed by other bat CoVs for novel disease emergence. Finally, the ability of SARS-like CoVs to be transmitted between and establish in new species (i.e., to undergo host shifts) is consistent with other CoVs. This has been shown for several CoVs of livestock species (*35*) and has been used to support their inclusion as 1 of the groups of viruses most likely to be responsible for emerging zoonoses, even before the emergence of SARS (*36*).

The total diversity of CoVs (including SARS-like CoVs) in bats has likely not been fully described. The genus *Rhinolophus* (*8*) contains 77 bat species distributed in Asia, Europe, and Africa. The recent discoveries of bat CoVs in the United States (*37*) and SARS-COVs in African bats (*38*) support the hypothesis that CoVs are diverse and widespread in bat species. Given the diversity of CoVs in this group, and their propensity for host shifts, further viral discovery in rhinolophids may assist in understanding and ultimately controlling the emergence of zoonotic viruses. Bats are increasingly recognized as reservoirs of many highly lethal zoonotic agents (*32*). Understanding their diversity, behavior, and mechanisms of virus transmission may play a key role in preventing future outbreaks of both known and unknown zoonotic diseases of bat origin.
